# TCR-Like CAR-T Cells Targeting MHC-Bound Minor Histocompatibility Antigens

**DOI:** 10.3389/fimmu.2020.00257

**Published:** 2020-02-28

**Authors:** Yoshiki Akatsuka

**Affiliations:** Department of Immunology, Nagoya University Graduate School of Medicine, Nagoya, Japan

**Keywords:** minor histocompatibility antigen, TCR-like antibody, adoptive immunotherapy, allogeneic stem cell transplantation, chimeric antigen receptor (CAR) cell

## Abstract

Minor histocompatibility antigens (mHAgs) in allogeneic hematopoietic stem cell transplantation are highly immunogenic as they are foreign antigens and cause polymorphism between donors and recipients. Adoptive cell therapy with mHAg-specific T cells may be an effective option for therapy against recurring hematological malignancies following transplantation. Genetically modified T cells with T cell receptors (TCRs) specific to mHAgs have been developed, but formation of mispaired chimeric TCRs between endogenous and exogenous TCR chains may compromise their function. An alternative approach is the development of chimeric antigen receptor (CAR)–T cells with TCR-like specificity whose CAR transmembrane and intracellular domains do not compete with endogenous TCR for CD3 complexes and transmit their own activation signals. However, it has been shown that the recognition of low-density antigens by high-affinity CAR-T cells has poor sensitivity and specificity. This mini review focuses on the potential for and limitations of TCR-like CAR-T cells in targeting human leukocyte antigen–bound peptide antigens, based on their recognition mechanisms and their application in targeting mHAgs.

## Introduction

Minor histocompatibility antigens (mHAgs), which are generated from polymorphic genes between a donor and recipient, are presented in the groove of human leukocyte antigen (HLA) molecules. In recipients undergoing allogeneic hematopoietic stem cell transplantation (allo-HSCT), mHAgs are recognized by donor T cells (1) and are highly immunogenic in the graft-vs.-host direction (2). Detection of T cell responses to molecularly defined and well-characterized mHAgs following allo-HSCT is possible through use of an HLA multimer reagent that incorporates the defined epitope peptide (3). In the context of hematologic malignancies, the therapeutic potential of T cells specific to mHAgs presented predominantly or exclusively on recipient target hematopoietic cells (including leukemia cells) but not on non-target non-hematopoietic cells has been shown via the graft-vs.-leukemia effect following donor lymphocyte infusion against recurring hematological malignancies (2–4). In addition, some mHAgs such as HA-1 and BCL2A1 have been found expressed in solid tumors, supporting the clinical applicability of immunotherapy in the allo-HSCT setting (5, 6). However, it is not always possible to selectively expand mHAg-specific T cells for their use in adoptive immunotherapy, primarily because of the cumbersome and time-consuming *in vitro* expansion procedure, which sometimes results in T cell exhaustion (7, 8). To overcome this problem, viral vectors encoding T cell receptor (TCR) α and β chain cDNAs cloned from high affinity mHAg-specific T cells have been used to genetically modify and redirect T cells toward the targeted mHAg (9, 10). Indeed, these so-called “TCR-T” cells have been shown to acquire the conferred antigen specificity, but mispairing between the introduced and endogenous TCR chains occasionally results in unwanted or unpredictable T cell specificities (11). Competition for CD3 complexes also leads to decreased signal transduction necessary for T cell function and proliferation. Various countermeasures have been devised to address these problems, including (1) the use of constant domains from other species such as mice (12), (2) introduction of disulfide or other bonds between the α and β TCR chains (13), (3) silencing of endogenous mRNA encoding TCR using siRNA (14), and (4) knockout of the TCR gene by means of gene editing technologies (15). An alternative approach was the development of chimeric antigen receptor (CAR)–T cells with TCR-like specificity, whose transmembrane and intracellular domains do not compete with endogenous TCR for CD3 complexes. This mini review will focus on the potential and limitations of applying TCR-like CAR-T cell technology to target HLA-bound mHAgs.

## Background of TCR-Like Antibodies and Their car-T Form

Recently, CD19-specific CAR-T cell therapies have been introduced in clinical practice with great success. Although clinical trials of CAR-T cells targeting promising candidate antigens other than CD19 are underway, the number of ideal tumor-specific targets is limited by the number of tumor-specific “cell-surface” antigens that are targetable with conventional monoclonal antibodies. By contrast, most potential tumor-specific antigens, such as proteins involved in cell proliferation and survival, are located in the intracellular region; there, they are degraded by proteasomes and may be displayed as antigenic peptides on major histocompatibility complex (MHC) class I and MHC class II molecules. These MHC-bound antigens are recognized by T cells with specific TCRs under physiological conditions. The affinity of the relevant TCRs is generally moderate or low because most tumor antigens are shared with normal cells but are generally overexpressed. Thus, reactive T cells with high-affinity TCRs undergo negative selection in the thymus (16). To target MHC-bound antigens, enhancement of TCR affinity with amino acid substitutions or development of a new mode of antibodies specific for peptide/MHC (pMHC) complexes is necessary. The latter are called TCR-like or TCR-mimic antibodies and can be used to redirect T cells to target antigens. The first TCR-like antibody was developed in 1982 to target the influenza PR8 antigen presented on murine H-2K^b^ (17), and the first attempt to construct CAR-T cells with a TCR-like antibody against MAGE-A1 presented on HLA-A1 was conducted in 2001 (18).

Initially, to generate TCR-like antibodies, a hybridoma-based method was used in which animals (mostly mice) were immunized with cells expressing pMHC or recombinant pMHC proteins. Then, sensitized splenic B cells were isolated and fused with a non-secretory myeloma cell line, which resulted in the generation of hybridoma cells, each of which produced monoclonal antibodies. New technology able to synthesize recombinant pMHC monomers or multimers (19) contributed to the preparation of antigens required not only for immunization, but also for the screening of hybridoma libraries. Antibodies raised by hybridoma-based methods consist of naturally selected light and heavy chain pairs; they possess a high affinity but have a limited repertoire of diversity because the pool size is restricted by the number of initial splenic B cells in the immunized mice.

In contrast, new phage library-based methods (20) utilize phages carrying randomly combined variable regions from light and heavy chains that have been amplified from a B cell pool. Their diversity size is approximately 10^9^–10^10^. It is possible to screen phages by positive and negative selection with target antigens and non-target antigens under various stringency conditions, as the fused variable genes in the recombinant phage genome are displayed as single-chain antibodies on their phage surface. Because most procedures can be performed with biochemical assays, this approach is robust and cost-/time-effective. However, the random recombination of variable regions from irrelevant light and heavy chains sometimes leads to antibodies with off-target binding capacity in addition to the desired pMHC specificity. Thus, careful and thorough screening in a wide array of normal tissues is necessary.

**Figure 1:**
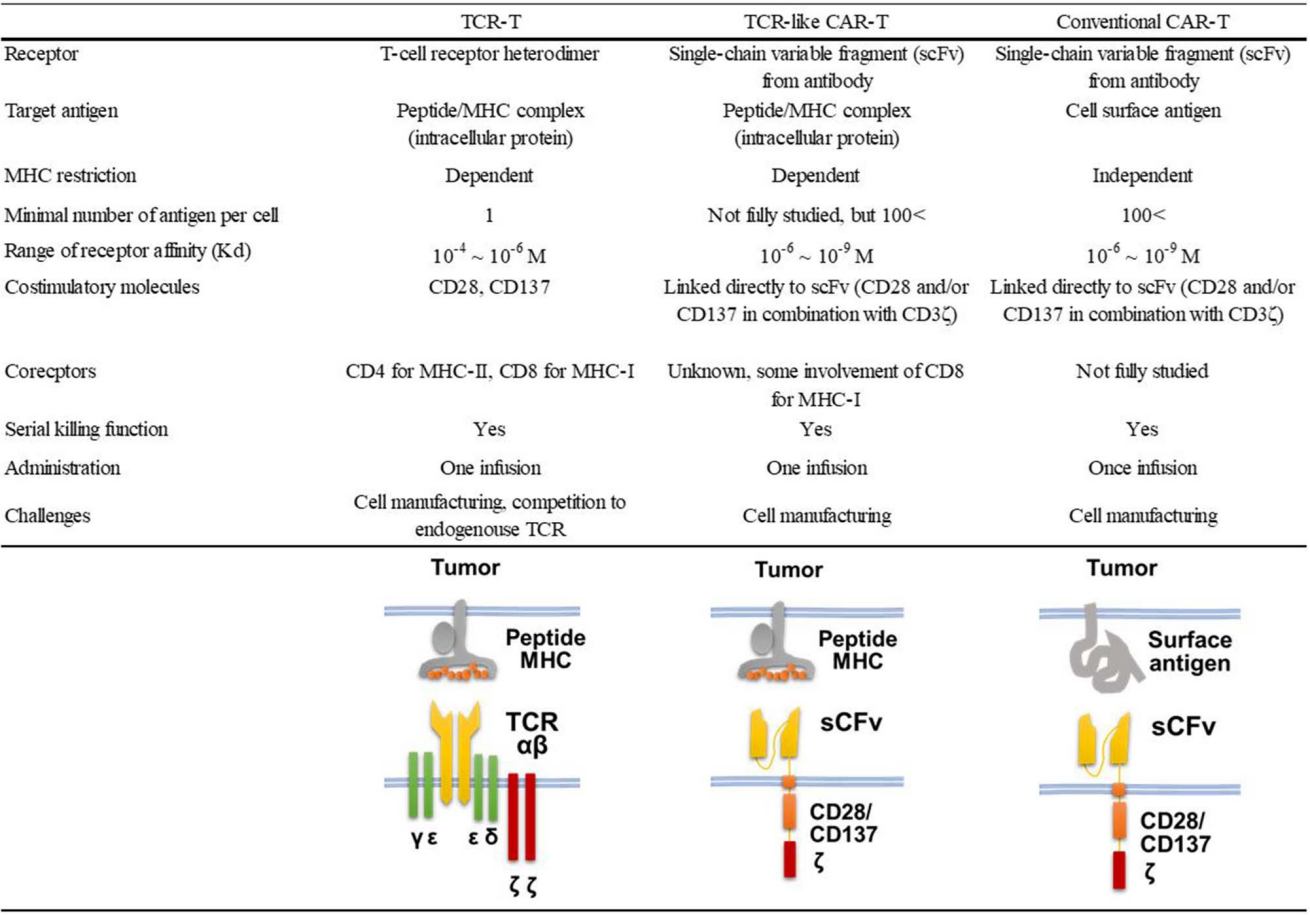
Characteristics of TCR-T and TCR-like CAR-T cells.

Nearly half of the reported TCR-like antibodies have been generated by the phage-based method (21). Among these, only 11 reports, including ours (22), described the application of TCR-like antibodies to CAR-T cell development (18, 23–32). As shown in Table 1, 10 of 12 such CAR-T cells targeted non-mutated antigens highly expressed in tumor cells, but none of them have been evaluated in clinical trials so far. All but one study that targeted the insulin-derived peptide presented on MHC class II used phage libraries whose clone size ranged from 2.85 × 10^8^ to 9 × 10^10^ clones. The dissociation equilibrium constant (K_D_) of their binding moiety ranged widely from 0.03 to 400 nM. This is in marked contrast to TCRs, the affinities of which generally range between 1 and 100 μM (33). Thus, natural TCR affinity is approximately 10^3^ to 10^5^ times weaker than those of the TCR-like antibodies. In addition, it has been shown that the use of the antibody's binding moiety as the antigen recognition domain of CARs can increase effector function, leading to the eradication of tumor cells with downregulated antigen expression at a level of 200 copies/cell (34). A similar density threshold of 300 copies/cell was reported for murine CAR-T cells targeting the glycoprotein OTS8 induced by cancer-specific mutation (35). As mentioned earlier, TCR gene-modified T (TCR-T) cells have also been developed since the first attempt by Heemskerk et al. (9). One phase I trial for TCR-T cells targeting HA-1 mHAg is currently underway (NCT03326921). The similarities and differences among TCR-like CAR-T cells, TCR-T cells, and conventional CAR-T cells are shown in Figure 1.

**Table 1:** List of TCR-like CAR-T cells.

**Antigen**	**MHC**	**Epitope**	**Target disease**	**Method**	**Library size**	**Immunization**	**Screening**	**Clone**	**Affinity (K_**D**_)**	**CAR signaling domains**	**References**
MAGE-A1	HLA-A1	EADPTGHSY	Cancer	Phage	3.7 × 10^10^	–	pMHC	G8	250 nM	CD4–FcεRIγ	(18)
MAGE-A1	HLA-A1	EADPTGHSY	Cancer	Phage	3.7 × 10^10^	–	pMHC	G8 Hyb3	250 nM 14 nM	CD28–FcεRIγ	(23)
NY-ESO-1	HLA-A*02:01	SLLMWITQC	Cancer	Phage	1.45 × 10^10^	–	pMHC	T1	2–4 nM	CD28–CD3ζ	(24)
WT1	HLA-A*02:01	RMFPNAPYL	Cancer	Phage	3.7 × 10^10^	–	pMHC	F2 F3	400 nM 30 nM	CD28- FcγRIγ	(25)
HMHA1	HLA-A*02:01	VLHDDLLEA	Blood cancer	Phage	5 × 10^8^	+	pMHC	#131	19.9 nM	CD28–CD3ζ	(22)
GP100	HLA-A*02:01	ITDQVPFSV	Melanoma	Phage	5.4 × 10^8^	–	pMHC	GPA7	183 nM	CD3ζ	(26)
WT1	HLA-A*02:01	RMFPNAPYL	Cancer	Phage	2.85 × 10^8^	–	pMHC	Q2L	3 nM	CD137–CD3ζ	(27)
AFP	HLA-A*02:01	FMNKFIYEI	Hepatoma	Phage	9 × 10^10^	–	pMHC	ET1402L1	0.03–0.2 nM	CD28–CD3ζ	(29)
WT1	HLA-A*02:01	RMFPNAPYL	Cancer	Phage	7 × 10^10^	–	pMHC	ESK1	0.1 nM	CD28–CD3ζ	(30)
WT1	HLA-A*24:02	CYTWNQMNL	Cancer	Phage	NA	–	pMHC	#213	741 nM	CD3ζ-CD357	(31)
Insulin	H-2 I-A^g7^	B:9–23 peptide	Autoimmune diabetes	Hybridoma	850	+	pMHC	mAb287	120 nM	CD28–CD3ζ, CD28–CD137–CD3ζ	(32)

## Affinity of TCR-Like Antibody and Density of Target pMHC

While the target antigen density per tumor cell in antibody-targetable tumors has not been measured in detail, the proteins CD19 and CD20 present on B cells targeted by therapeutic antibodies and CAR-T cells are relatively well studied; for example, the antigen density of CD19 has been reported to be 2 × 10^4^ to 3 × 10^4^ molecules/cell in normal B cells and 0.5 × 10^4^ to 3 × 10^4^ molecules/cell in malignant B cells (36, 37). In contrast, despite the fact that the affinities of canonical native TCRs are 3 to 5 logs lower than those of the conventional antibodies used in CD19 CAR-T cells, it is interesting that T cells can recognize pMHCs presented on cell surfaces at much lower densities. Several studies have demonstrated that the minimum number of pMHC complexes required to activate T cells is <10 per cell, although this number depends on the presence of coreceptors and the status of the cell (38–40). Using comprehensive mass spectrometry analysis of a peptide pool stripped from MHC molecules, it has been shown that certain peptides are expressed at a frequency of 100 to 10,000 copies/cell, which corresponds to 0.1 to 10% of peptides presented by one kind of MHC allele, such as H-2 K^b^ or D^b^ in mice (38). Assuming the number of each MHC class I molecule per cell is 1 × 10^5^ to 2 × 10^5^ and the average copy number of a given peptide is 200, then 500–1,000 peptides with different sequences are expressed on one kind of MHC per cell (38). However, peptides with much lower densities must be expressed at a much wider variety and still be recognized by relevant T cells.

In terms of human mHAgs, it has been shown that HLA-A^*^02:01–restricted HA-1^H^ is present at 80 copies/cell, while its counterpart HA-1^R^ is <5 copies/cell because of its 27-fold lower affinity to the HLA-A2 molecule (41, 42). The other HLA-A^*^02:01–restricted HA-2^M^ mHAg was found to be present below the detection limit of 0.04 to 0.2 copy/cell (43). It is speculated that these cytotoxic T lymphocytes (CTLs) possess high-affinity TCRs because mHAgs are non–self-antigens similar to pathogens (44), and thus no thymic or peripheral tolerances affect T cells (16). Given that CTLs specific for HA-1^H^ and HA-2^M^ were readily detected at a range of 0.21 to 1.57% among CD8^+^ cells in patients receiving allogeneic HSCT and donor lymphocyte infusion and that the sorted T cells showed specific killing activity against mHAg-positive target cells (3), it is clear that T cells should have at least two modes of action when recognizing antigens via canonical (cognate) TCR moieties vs. CAR moieties.

A small number of pMHC complexes can serially engage and trigger up to approximately 200 TCRs (45). Additionally, efficient T cell activation occurs within an optimal dwell-time range of TCR–pMHC interaction using MHC with mutations in its antigen-binding site (46). This is thought to be possible by a TCR–pMHC engagement of moderate affinity rather than super-high affinity as seen in antibody–antigen binding. Furthermore, it has been shown that CD20 CAR-T cells require approximately 15,000 CD20 molecules per target cell to trigger 10,000 CAR molecules per T cell, suggesting that a decreased number of triggered CAR molecules are necessary because of a lack of serial engagement (47). In contrast, decreased signaling and effector function did not occur when high-density antigens were present on the target cells (48). These observations shed light on the design of CAR-T cells equipped with TCR-like antibodies.

## Considerations Toward Car-T Cells Equipped With TCR-Like Antibodies

Attempts to generate CAR-T cells possessing a TCR-like antibody moiety (TCR-like CAR-T) have been challenging, insofar as target cells express a very low density of pMHC. Furthermore, it has not been clarified whether a “serial engagement” scenario can occur even in the case of TCR-like CAR-T cells with a TCR-like antibody moiety that has low affinity comparable to canonical TCR. To this end, fine tuning of the TCR-like antibody moiety is crucial. Crystal structural analysis revealed that TCRs bind in a conserved diagonal mode (33); thus, some guidelines for tuning their affinity either to the epitope peptide or to an MHC scaffold have been devised. Alternatively, TCR-like antibodies take various binding modes, and their fine tuning is limited to the complementary determining region 3 (49, 50).

Researchers have attempted to ensure the specificity of modified antibodies in targeting amino acids among an array of peptides presented on a single restriction MHC molecule. This is critical because expression as a CAR-T form on the T cell surface, where other adhesion molecules and coreceptors are aligned, may further modify the functional avidity of CAR-T cells. Akahori et al. (31) comprehensively analyzed their low-affinity TCR-like CAR-T cells specific to the WT1_235−243_ peptide presented on HLA-A^*^24:02 molecules by incorporating (1) alanine substitution analysis of the epitope peptide to identify both the amino acid residues that trigger interaction with TCR CAR-T cells and those used for anchors; (2) *in silico* searches for potentially cross-reactive peptides that contain the predetermined contact amino acid residues in their sequence, followed by *in vitro* assays to test their potential to stimulate TCR CAR-T cells; and (3) *in vitro* cross-reactivity assays against other HLA molecules using a panel of cell lines. Their TCR-like antibody (clone #213) has a K_D_ of 741 nM (31), which is close to the lowest natural TCR affinity range of 1 to 100 μM (33). This strategy may contribute to the sufficient functional avidity (here, a biological readout reflecting T cell responsiveness *in vitro*) and retained specificity of their TCR-like CAR-T cells, although the WT1_235−243_ peptide density on WT1- and HLA-A24–positive cells has not been determined to date. It has been shown that two kinds of conventional CAR-T cells, with K_D_ values of 1 nM and 1,616 nM to the same extracellular domain of HER2 molecule, had comparable lytic activity against target cells with high HER2 expression; however, CAR-T cells with low affinity showed more efficient lytic activity against target cells with limited HER2 expression (51).

Of additional concern are on-target/off-tumor and off-target toxicities. Such toxicities have been observed in adoptive immunotherapy trials using affinity matured TCR-T cells specific to MAGEA3 (52) or CAR-T cells specific to CA9 (53) or CEA (54), all of which are expressed in normal tissues at very low levels. Oren et al. (25) demonstrated that their TCR-like CAR, which had an elevated receptor affinity (30 nM) compared with that of others (Table 1), results in some loss of specificity and decreased cell survival when transduced into HLA-A2–positive but HLA-A2–negative T cells. This may be due to fratricide, wherein a high-affinity antibody cross-reacts with non-target peptides presented on coexisting T cells. A similar phenomenon has been reported in which the addition of an anti-CD38–blocking antibody saved CD38 antibody-equipped CAR-T cells from fratricide, as CD38 is dimly expressed on T cells (55). To prevent these toxicities in future clinical studies, a systematic screening system for cross-reactivity testing must be devised. A humanized mouse model, where HLA-matched tumor cells and immune cells from the patient are engrafted into an HLA-transgenic non-obese diabetic/severe combined immune-deficient/common gamma chain knockout mouse, may serve as a screening platform (56, 57).

## Application of TCR-Like Car-T Cells to MHAGS

Inaguma et al. (22) first included a TCR-like antibody against the HA-1^H^ mHAg in CAR-T cell preparations (Table 1). Using a phage library prepared from splenic B cells isolated from HLA-A2–transgenic mice immunized with HA-1^H^/HLA-A^*^02:01 tetramers, specific single-chain antibodies were isolated by multiple rounds of panning. HLA-A2 transgenic mice were used to omit xenogeneic immune responses against human HLA-A2 molecules. Although a resulting CAR-T cell (clone #131) with high affinity binding (K_D_ = 19.9 nM) was stained with HLA-A2/HA-1 tetramers with an intensity equivalent to cognate cytotoxic T cell clones, the CAR-T cell required 100-fold higher peptide density to exert cytotoxic function (22). Another clone (#9) with moderate to low affinity (K_D_ = 446 nM) was also tested, and researchers found that its CAR-T form exhibits ~10-fold increased activity, supporting the observations by Akahori et al. (31).

Major histocompatibility complex class I–restricted autosomal mHAgs are generated by various molecular mechanisms (2, 58). The majority of mHAgs are generated by single-nucleotide substitutions that engender amino acid substitutions, whereas others are generated by frameshift mutations or whole gene deletions (e.g., UGT2B17) (59). Among these, allelic variant peptides of mHAgs such as ACC-1 (60, 61) and HB-1 (62, 63) are expressed with an affinity similar to that of their restriction HLA molecules. Generating TCR-like antibodies to these mHAgs may be difficult, as the difference between two allelic variants is a single amino acid, and TCR-like antibodies may bind to more than one (e.g., three) amino acid in the peptide (31). Given this issue of specificity, it is more reasonable to target mHAgs in which only one allelic variant is exclusively or at least highly expressed. Because the HA-1^R^ peptide cannot be presented on the cell surface (42), HA-1^H^ is an ideal target. Other mHAgs resulting from frameshift mutations due to various polymorphisms, such as LRH-1 (64), HMSD (65), or PANE1 (66), may be suitable, as the donor-recipient pair possesses different amino acid sequences at the corresponding positions (or a null peptide in the case of gene deletion or miss-sense polymorphisms). Finally, target mHAgs must be hematopoietic system–specific to avoid graft-vs.-host disease.

In the production of antibodies, both major methods (hybridoma and phage library) have advantages and disadvantages. Although it is thought that naturally occurring antibodies with TCR-like specificity are extremely rare, with the exception of those against mHAgs encoded on the Y chromosome (H-Y antigen) (67), such antibodies can be present in patients receiving mHAg-mismatched HSCT. If this is the case, pooled B cells from such patients may serve as a source for phage display library. Alternatively, immunization with mHAg-pMHC complexes from HLA-transgenic mice (22) may also be a source.

## Conclusion

Based on the state of the field, the generation of TCR-like CAR-T cells that use an all-in-one chimeric receptor equipped with modifiable intracellular signaling domains that can be applied quickly to patients as adoptive cell therapy is of utmost interest. Chimeric antigen receptor–T cells have a strong advantage over modified TCR gene–introduced T cells because they have a built-in signaling domain, which works even in the absence or downregulation of costimulatory signals from target cells. However, various improvements in TCR gene–introduced T cells make this option safer and more promising than are CAR-T treatments (10). In any case, the establishment of robust and efficient screening systems, including a variety of panel peptides, HLA-typed cell lines, and animal models for the evaluation of TCR-like antibody efficacy and toxicity, as well as the careful planning of preclinical experiments, is necessary for obtaining TCR-like antibodies with potential clinical applications. In contrast to passive immunotherapies such as gene-modified T cells, active immunotherapies such as peptide (68, 69) or DNA vaccinations with or without adjuvants using dendritic cells are being tested in clinical trials (2). These approaches are thought to be more feasible and less expensive, as gene-modified cells are under strict regulations. However, it is too early to compare the two major approaches as only limited phase I/II clinical data have been publicly reported, including a phase I dose evaluation study for an HA-1 mHAg vaccine (70). Further studies for individual interventions are necessary to define the optimal methods and patient populations for mHAg-targeted immunotherapy.

## Author Contributions

The author confirms being the sole contributor of this work and has approved it for publication.

### Conflict of Interest

The author declares that the research was conducted in the absence of any commercial or financial relationships that could be construed as a potential conflict of interest.
